# Treatment of Bacteriuria by Internal Medication

**Published:** 1908-08

**Authors:** 


### Treatment of Bacteriuria by Internal Medication.

J. W. Churchman (*Johns Hopkins Hospital Reports*, Vol. 13, p. 139|207), says his experience demonstrates that helmitol is not superior to urotropin in point of efficacy; in the cases in which urotropin had not wholly freed the urine from bacteria, resort to helmitol also proved unavailing. Regarding the prevention of bacteriuria, Churchman says that in no cystoscopy done in the practice of Dr. Young, on patients whose urine was uninfected and who had received urotropin internally, did the subsequent examination of the urine by the centrifugalisation method reveal organisms. But in one case in which the patient had not received this prophylactic medication, an infection did develop. "Clinical evidence, apart from the corroborating evidence of experimental work, very strongly suggests the value of drugs internally for prophylaxis of bacteriuria; such use not only is indicated, but may confidently be expected to be efficacious."

Incipient bacteriuria without cystitis, as clinical evidence goes to show, can in the majority of cases be inhibited by internal medication. In exceptional instances the bacteriuria will persist despite treatment. In the majority of bacteriurias associated with cystitis, as clinical evidence goes to show, it is practically impossible to wholly remove the organisms which have gotten a sufficient hold on the bladder to produce cystitis. Pus may diminish in amount, the symptoms be relieved, and the urine cleared; but only occasionally will the infection completely disappear.

From a series of bacteriological experiments with the urines of patients who took urotropin, methylene blue or salol by mouth, Churchman concludes:

1. Administration of urotropin, methylene blue or salol renders the urine inhibitive of the growth of the staphylococcus pyogenes, streptococcus pyogenes, B. typhosus, B. coli communis and B. proteus vulgaris.

2. Urotropin and methylene blue are more markedly efficacious (inhibitive) than salol; the choice lies with the first.

3. These drugs effect inhibition of bacterial development rather than destruction of bacterial life. They render urine an uncongenial medium for growth, but not an environment necessitating death.

4. Their effect is weakest on the staphylococcus pyogenes and strongest on the B. typhosus and streptococcus pyogenes.

---

Professor Crossen in his Diagnosis and Treatment of Diseases of Women (Mosby Med. Book & Pub. Co., St. Louis, 1907) points out that when uterine suppositories are used, more will be accomplished by incorporating into them some penetrating antiseptic, such as collargol or Credé's ointment, than by the use of the surface antiseptics usually employed.

